# Texas State Board of Health, Austin

**Published:** 1916-09

**Authors:** 


### Texas State Board of Health, Austin.

AN ACTUAL DEMONSTRATION IN PUBLIC HEALTH WORK.

For many years the agricultural departments have been demonstrating the advantage of scientific farming by establishing demonstration farms in different points in the counties of Texas. At these farms the soil, the climate, the rainfall and all conditions affecting agriculture, are studied.

THE SAME PLAN HAS BEEN ADOPTED IN PUBLIC HEALTH WORK.

In Leon, Harris and Polk counties three demonstration districts in each county will be selected. Every feature affecting the health of the inhabitants of the districts will be studied.

The plan of this intensive health work begins with a series of lectures on the prevention of sickness, delivered as nearly as possible in every locality in the county to both whites and blacks. Upon the interest shown in these lectures will depend the selection of the three demonstration districts. Each district will include approximately 1000 people, and will be typical of the conditions in the county.

When districts are selected a sanitary survey will be made, showing on the map every road, home, creek, privy, well and every disease-producing condition. A laboratory will be established, a house-to-house canvass will be made, the home and its surroundings, as well as public buildings, inspected, and all unsanitary conditions pointed out.

The school children will be inspected for physical defects, and where such defects are found, cards will be sent to the parents, explaining any defect, and the necessity for correcting same.

During the demonstration work lecture with stereopticon views will be given on malaria, hookworm, typhoid, tuberculosis, and other preventable diseases, as well as home and municipal sanitation, and the prevention of infant mortality.

During the last weeks of the compaign carpenters will be secured and with the assistance of the heads of the families, houses will be screened and sanitary privies built.

With malaria, hookworm and typhoid eradicated, the value of many farms, now almost untenable, will be increased in value several times, not to mention the saving in *human lives*.

This campaign will be under the direction of Dr. P. W. Covington, of the International Health Commission, working under the auspices of the State Board of Health.

The Texas State Board of Health will be glad to answer any inquiry from those interested in such work.

